# Supplementary figures and images for: Selective activation of ipRGC modulates working memory performance
Source: PLoS One. 2025 Jun 30;20(6):e0327349. doi: 10.1371/journal.pone.0327349 (PMC12208440; doi:10.1371/journal.pone.0327349)

**A**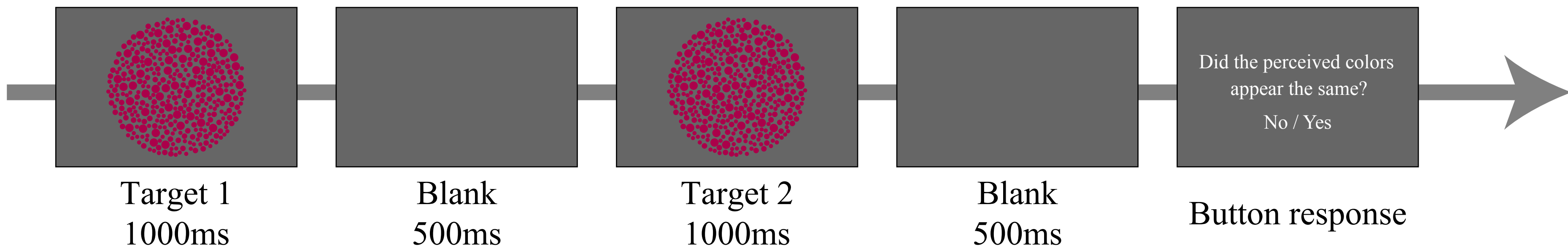**B**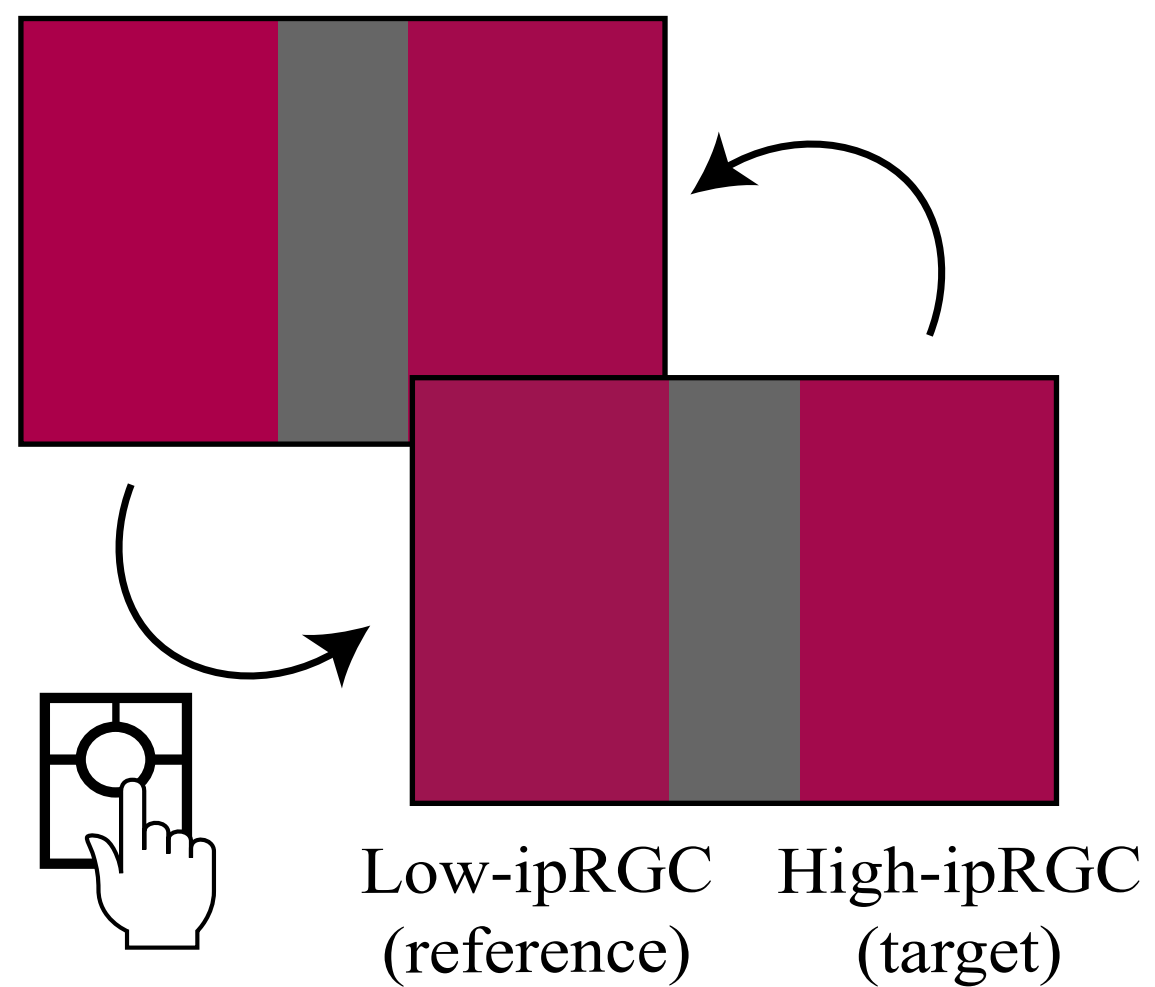**C**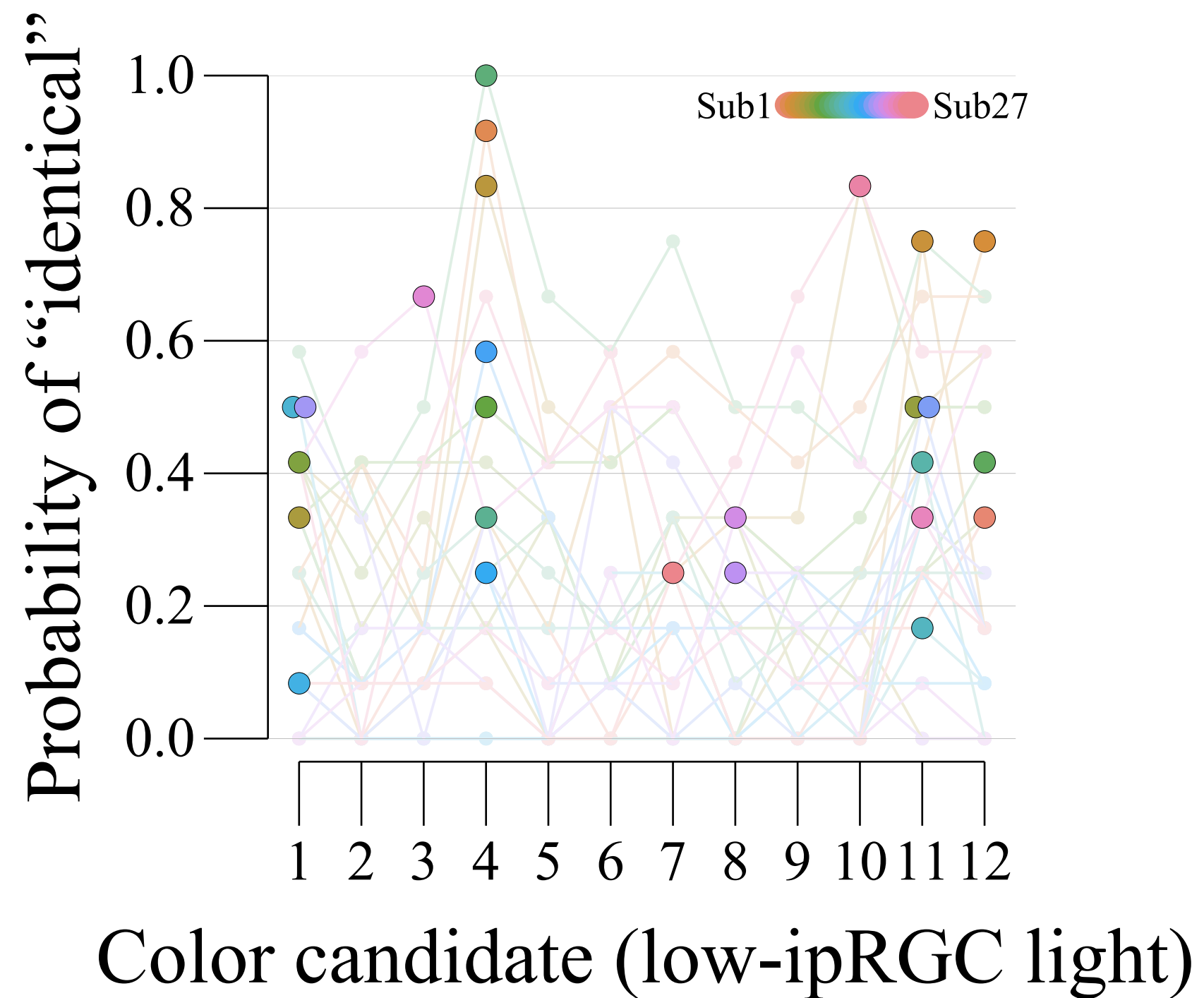

Supplement: S1 Fig — (A) Experimental design for stimulus tuning and (B) brightness evaluation (C) The response probability of “identical” in the stimulus tuning experiment. The bright circles are chosen as metamer light as low ipRGC condition for each subject as illustrated in different colors. (PDF) [file pone.0327349.s001.pdf]

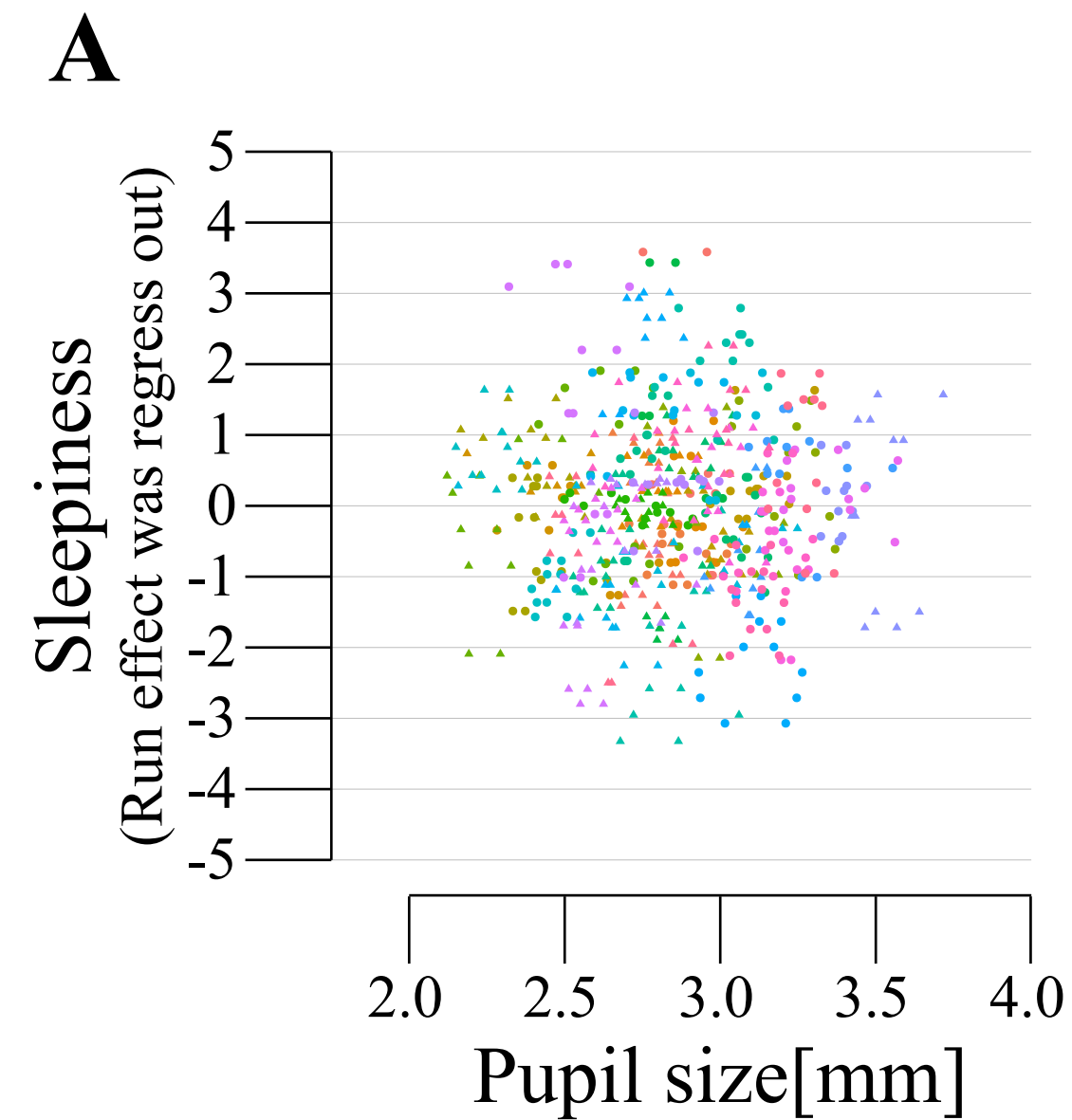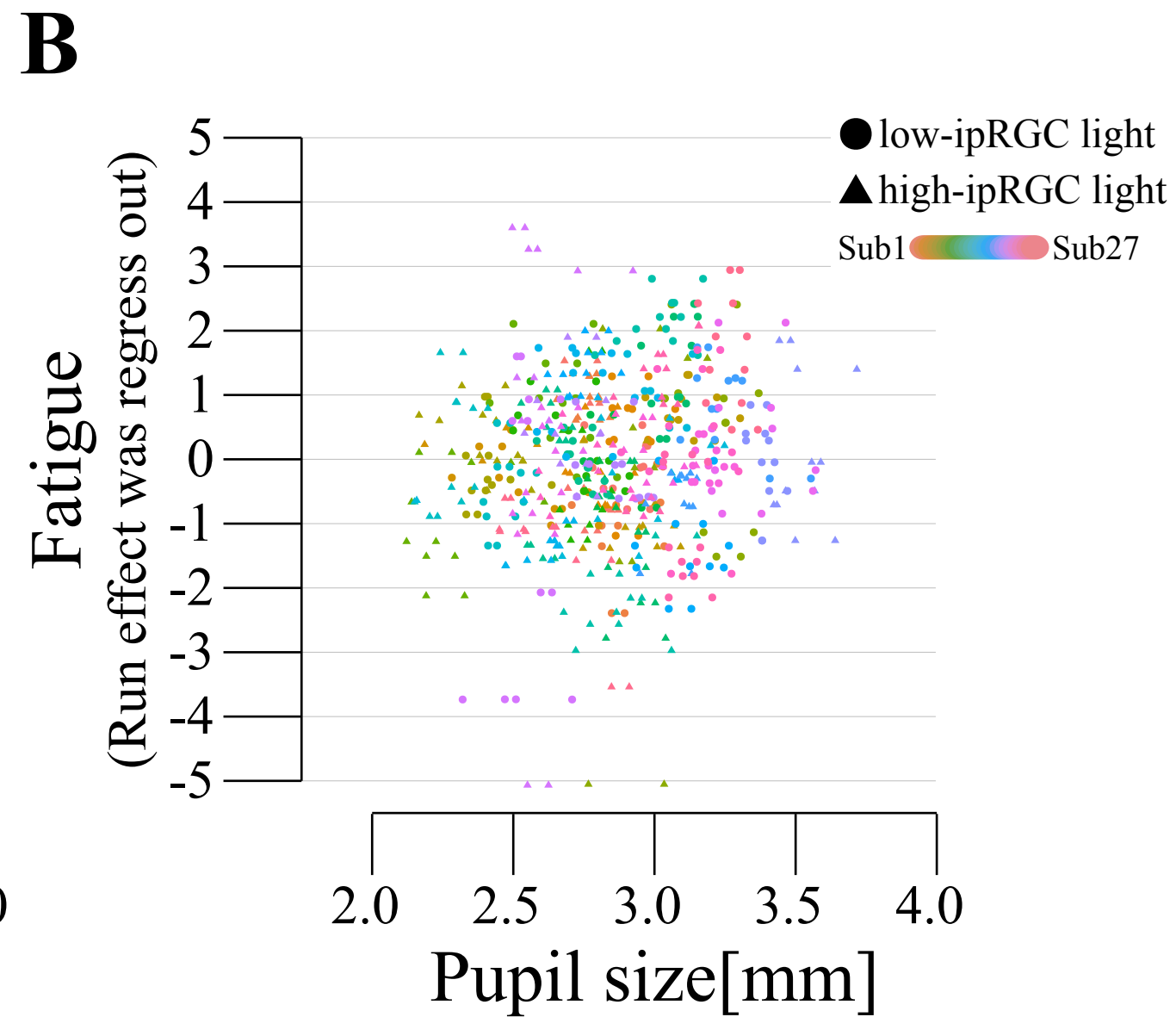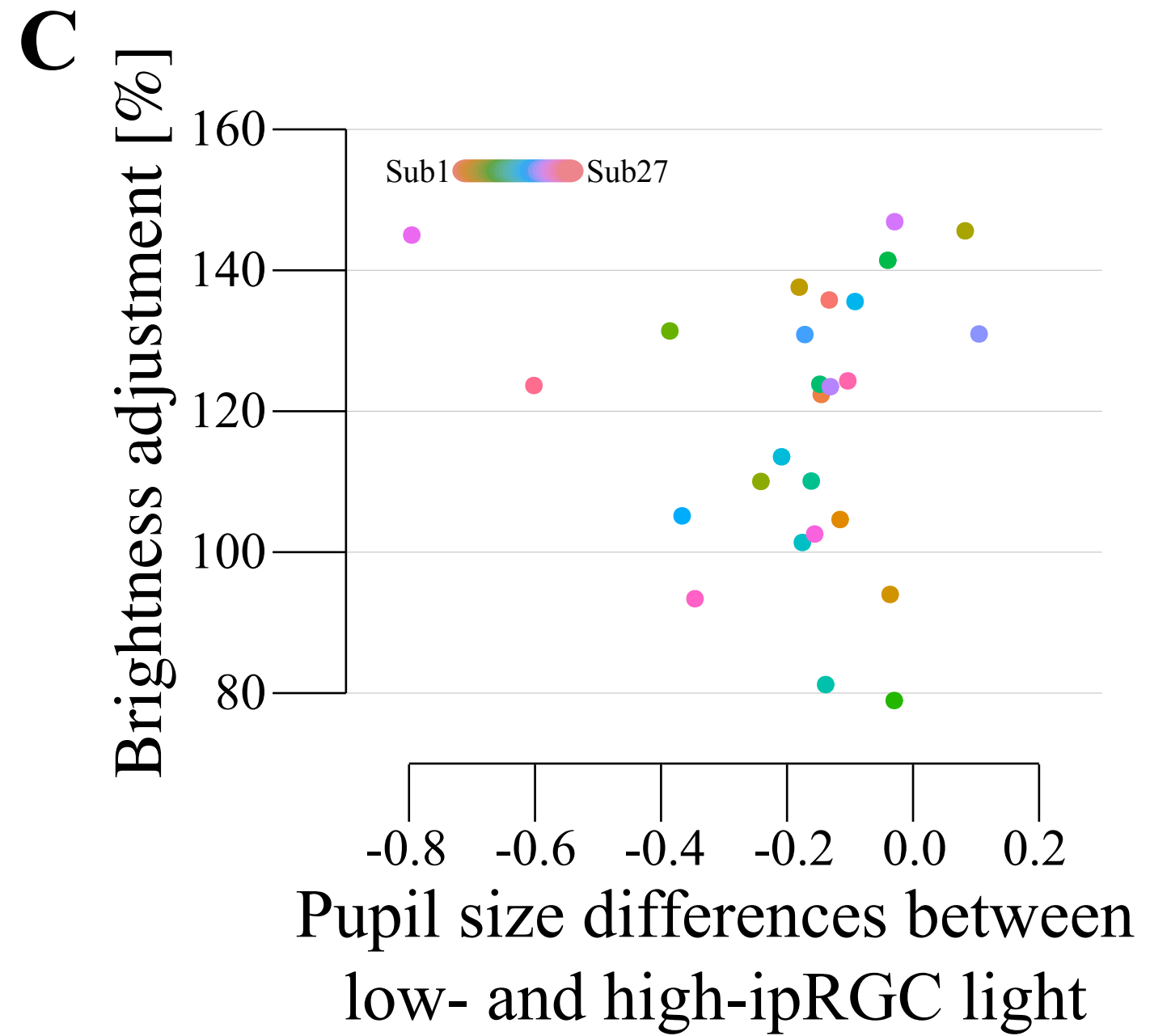

Supplement: S2 Fig — lot showing the relationship between pupil size and sleepiness ratings. (B) Scatter plot showing the relationship between pupil size and fatigue ratings. In both (A) and (B), circle markers represent data from the low-ipRGC light condition, and triangle markers represent data from the high-ipRGC light condition. (C) Scatter plot showing the relationship between the degree of pupil constriction under high-ipRGC light (horizontal axis) and the brightness adjustment values from the brightness-matching experiment (vertical axis). Each color represents data from an individual participant. (PDF) [file pone.0327349.s002.pdf]
