# Supplementary material for: Selective activation of ipRGC modulates working memory performance
Source: PLoS One. 2025 Jun 30;20(6):e0327349. doi: 10.1371/journal.pone.0327349 (PMC12208440; doi:10.1371/journal.pone.0327349)
Supplement: S1 Table — (DOCX) [file pone.0327349.s003.docx]

**Supplementary Materials for “Selective Activation of ipRGC Modulates Working Memory Performance”**

**Yuta Suzuki^1*^, Shigeki Nakauchi^2^, Hsin-I Liao^1^**

^1^ NTT Communication Science Laboratories, NTT Corporation, Atsugi, Kanagawa, Japan

^2^ Department of Computer Science and Engineering, Toyohashi University of Technology, Toyohashi, Aichi, Japan

^*^Corresponding author

E-mail: [suzukiy970@gmail.com](mailto:yuuta.suzuki.fc@hco.ntt.co.jp%22%20%5Ct%20%22_blank)

##

**
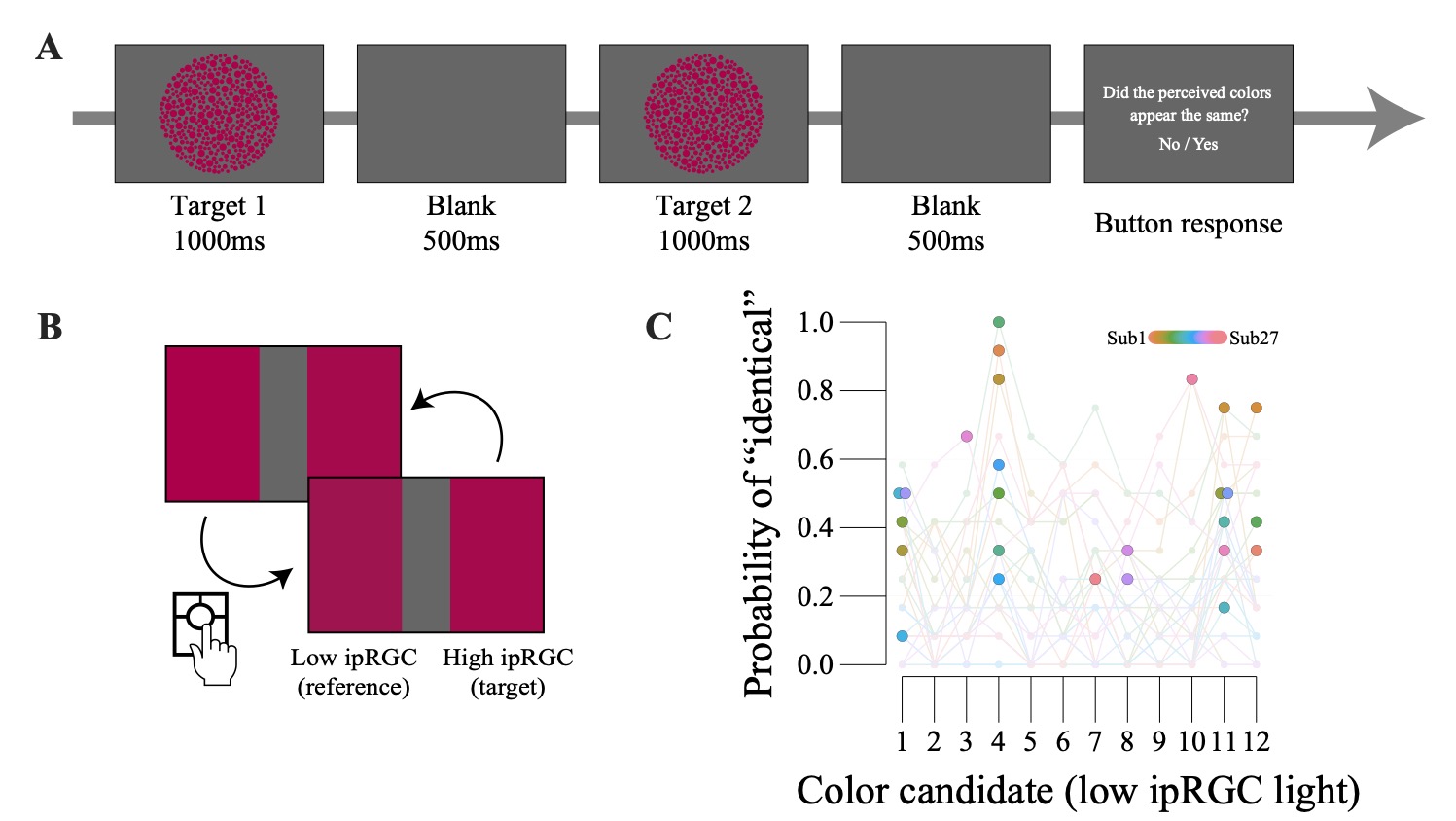
 S1 Fig. Experimental design for stimulus tuning and brightness experiment.** (A) Experimental design for stimulus tuning and (B) brightness evaluation (C) The response probability of “identical” in the stimulus tuning experiment. The bright circles are chosen as metamer light as low ipRGC condition for each subject as illustrated in different colors.

**S1 Table**. Hit, miss, false alarm (FA) and correct rejection (CR) rate in 1- and 2-back task.

|  |  | Low ipRGC | High ipRGC |
| --- | --- | --- | --- |
| 1-back | Hit (n.s.) | 95.93 ± 7.254 | 96.491 ± 4.977 |
|  | Miss (n.s.) | 0.041 ± 0.073 | 0.035 ± 0.05 |
|  | FA (n.s.) | 0.325 ± 0.456 | 0.366 ± 0.364 |
|  | CR (n.s.) | 99.675 ± 0.456 | 99.634 ± 0.364 |
| 2-back | Hit (*) | 88.754 ± 6.378 | 91.718 ± 4.996 |
|  | Miss (*) | 0.112 ± 0.064 | 0.083 ± 0.05 |
|  | FA (+) | 1.875 ± 1.403 | 1.373 ± 0.828 |
|  | CR (+) | 98.125 ± 1.403 | 98.627 ± 0.828 |

** S2 Fig. Correlations between pupil size and fatigue, sleepiness, and brightness adjustment.** lot showing the relationship between pupil size and sleepiness ratings. (B) Scatter plot showing the relationship between pupil size and fatigue ratings. In both (A) and (B), circle markers represent data from the low-ipRGC light condition, and triangle markers represent data from the high-ipRGC light condition. (C) Scatter plot showing the relationship between the degree of pupil constriction under high-ipRGC light (horizontal axis) and the brightness adjustment values from the brightness-matching experiment (vertical axis). Each color represents data from an individual participant.

To examine whether pupil size could be attributed to variations in subjective fatigue and sleepiness, we computed correlations between pupil size and these subjective ratings. A linear mixed-effects model was used, with pupil size as within-subject factors and subject as a between-subject factor, following the equation: $Rating\sim pupil size+\left( 1|Subject \right)$. We found the fatigue ratings, but not sleepiness ratings, were significantly correlated with pupil size (fatigue: $t$(25) = 2.819, $p$=0.005, $d_{z}$ = 0.231; sleepiness: $t$(25) = -0.205, $p$=0.838, $d_{z}$ = 0.017).

In addition, brightness adjustment values (i.e., how much brighter the high-ipRGC light appeared) could not be explained by pupil size ($t$(25) = -0.246, $p$=0.808, $d_{z}$ = 0.098). One possible interpretation is that distinct ipRGC subtypes contribute to different functional outcomes[1,2]. For example, M1-type ipRGCs, which primarily project to non-image-forming targets such as the SCN and OPN, may be more directly involved in pupil size. In contrast, non-M1 subtypes are known to project to image-forming regions such as the LGN and are implicated in visual processing such as brightness enhancement.

**References**

1. Hannibal J, Christiansen AT, Heegaard S, Fahrenkrug J, Kiilgaard JF. Melanopsin expressing human retinal ganglion cells: Subtypes, distribution, and intraretinal connectivity. J Comp Neurol. 2017;525: 1934–1961. doi:10.1002/cne.24181

2. Schmidt TM, Chen S-K, Hattar S. Intrinsically photosensitive retinal ganglion cells: many subtypes, diverse functions. Trends Neurosci. 2011;34: 572–580. doi:10.1016/j.tins.2011.07.001
