# Supplementary material for: Selective activation of ipRGC modulates working memory performance
Source: PLoS One. 2025 Jun 30;20(6):e0327349. doi: 10.1371/journal.pone.0327349 (PMC12208440; doi:10.1371/journal.pone.0327349)
Supplement: Attachment — Submitted filename: [R2]reviewer_report_ys_HL_ys.docx [file pone.0327349.s006.docx]

Reviewer 1’s comments:

Reviewer #1: I appreciate the authors for their work on the revised manuscript. I think this manuscript is almost ready to be published. I have two remaining points that I hope the authors can address before publishing this article.

We sincerely thank the reviewer for further feedback on our manuscript. Below, we address your two remaining points.

**Point 1.** Line 82, reference [22] and that sentence came out of nowhere. I will suggest the authors put this reference in the discussion section.

Thank you for your helpful suggestion. We have moved the sentence and reference to the Discussion section (Line 426-428), where it fits within the context of the content.

**Point 2.** From my understanding, partial eta-squared =0.14 is already a large effect size, I am not sure where and how the authors derived 0.3 still.

Thank you for pointing this out. You are absolutely right. To clarify, the effect size value of 0.3 we referred to during our power analysis is Cohen’s f, not partial eta-squared. We apologize for the misstatement in our earlier version. We have now corrected the manuscript to clearly state that we used an expected effect size of Cohen’s f = 0.3, which corresponds to $\eta_{p}^{2}$=0.083.
